# Fully Automated MR Based Virtual Biopsy of Cerebral Gliomas

**DOI:** 10.3390/cancers13246186

**Published:** 2021-12-08

**Authors:** Johannes Haubold, René Hosch, Vicky Parmar, Martin Glas, Nika Guberina, Onofrio Antonio Catalano, Daniela Pierscianek, Karsten Wrede, Cornelius Deuschl, Michael Forsting, Felix Nensa, Nils Flaschel, Lale Umutlu

**Affiliations:** 1 Department of Diagnostic and Interventional Radiology and Neuroradiology, University Hospital Essen, Hufelandstr. 55, D-45147 Essen, Germany; rene.hosch@uk-essen.de (R.H.); vicky.parmar@uk-essen.de (V.P.); Cornelius.Deuschl@uk-essen.de (C.D.); Michael.Forsting@uk-essen.de (M.F.); Felix.Nensa@uk-essen.de (F.N.); Nils.Flaschel@uk-essen.de (N.F.); lale.umutlu@uk-essen.de (L.U.); 2 Department of Neurology, Division of Clinical Neurooncology, University Hospital Essen, D-45147 Essen, Germany; Martin.Glas@uk-essen.de; 3 Department of Radiotherapy, University Hospital Essen, D-45147 Essen, Germany; nika.guberina@uk-essen.de; 4 Department of Radiology, Division of Abdominal Imaging, A. Martinos Center for Biomedical Imaging, Massachusetts General Hospital, Harvard University Medical School, Boston 02114, MA, USA; ocatalano@mgh.harvard.edu; 5 Department of Neurosurgery, University Hospital Essen, D-45147 Essen, Germany; Daniela.Pierscianek@uk-essen.de (D.P.); Karsten.Wrede@uk-essen.de (K.W.)

**Keywords:** multiparametric MRI, radiomics, cerebral glioma, radiomics-based phenotyping and tumordecoding

## Abstract

**Simple Summary:**

Over the past few years, radiomics-based tissue characterization has demonstrated its potential for non-invasive prediction of the genetic profile and grading in cerebral gliomas using multiparametric MRI. The aim of our study was to investigate the feasibility and diagnostic accuracy of a fully automated radiomics analysis based on a simplified MR protocol derived from various scanner systems to prospectively ease the transition of radiomics-based non-invasive tissue sampling into clinical practice. Using an MRI with non-contrast and post-contrast T1-weighted sequences and FLAIR, our workflow automatically predicts the IDH1/2 mutation, the ATRX expression loss, the 1p19q co-deletion and the MGMT methylation status. It also effectively differentiates low-grade from high-grade gliomas. In summary, the present study demonstrated that a fully automated prediction of grading and the genetic profile of cerebral gliomas could be performed with our proposed method using a simplified MRI protocol that is robust to variations in scanner systems, imaging parameters and field strength.

**Abstract:**

Objective: The aim of this study was to investigate the diagnostic accuracy of a radiomics analysis based on a fully automated segmentation and a simplified and robust MR imaging protocol to provide a comprehensive analysis of the genetic profile and grading of cerebral gliomas for everyday clinical use. Methods: MRI examinations of 217 therapy-naïve patients with cerebral gliomas, each comprising a non-contrast T1-weighted, FLAIR and contrast-enhanced T1-weighted sequence, were included in the study. In addition, clinical and laboratory parameters were incorporated into the analysis. The BraTS 2019 pretrained DeepMedic network was used for automated segmentation. The segmentations generated by DeepMedic were evaluated with 200 manual segmentations with a DICE score of 0.8082 ± 0.1321. Subsequently, the radiomics signatures were utilized to predict the genetic profile of ATRX, IDH1/2, MGMT and 1p19q co-deletion, as well as differentiating low-grade glioma from high-grade glioma. Results: The network provided an AUC (validation/test) for the differentiation between low-grade gliomas vs. high-grade gliomas of 0.981 ± 0.015/0.885 ± 0.02. The best results were achieved for the prediction of the ATRX expression loss with AUCs of 0.979 ± 0.028/0.923 ± 0.045, followed by 0.929 ± 0.042/0.861 ± 0.023 for the prediction of IDH1/2. The prediction of 1p19q and MGMT achieved moderate results, with AUCs of 0.999 ± 0.005/0.711 ± 0.128 for 1p19q and 0.854 ± 0.046/0.742 ± 0.050 for MGMT. Conclusion: This fully automated approach utilizing simplified MR protocols to predict the genetic profile and grading of cerebral gliomas provides an easy and efficient method for non-invasive tumor decoding.

## 1. Introduction

Recent developments in radiomics based image analysis have enabled new possibilities to non-invasively determine the histopathology and/or genetic profile of a number of different tumor entities [1]. This is especially important for cerebral gliomas, as the latest WHO classification of 2016 puts the focus on the genetic profile to classify tumors precisely and plan personalized therapy regimens accordingly [2]. While this change in tumor classification aims to improve patient management and treatment, it implies the requirement for invasive tissue sampling to the detriment of potentially severe biopsy and/or surgical complications, resulting in a mortality rate of 2.8% [3]. As a result, there is a high demand for non-invasive tissue analysis to initiate adequate therapy regimens without potential biopsy-associated side effects. In particular, this is true for inoperable tumors that would be solely biopsied. Over the past few years, a number of studies demonstrated the great potential of MRI-based image analyses for the tumor decoding of gliomas [4]. Here, various approaches to image analysis for predicting the genetic profile of gliomas have emerged in recent years [5,6,7]. On the one hand, radiomics features can be extracted from segmentations and be used to train machine learning algorithms [8,9,10,11]. On the other hand, the features can be extracted from a segmentation using convolutional neural networks. Here, Guta et al. were able to show that features extracted using CNN lead to better predictions for the grading of cerebral gliomas than features extracted using Radiomics [5]. Third, various networks have been established for direct image analysis of cerebral gliomas utilizing a variety of deep learning networks [6,12]. All approaches come with their own advantages and disadvantages. While the traditional approach with radiomics has traceable features, allowing them to be replicated in studies [13], the segmentation required and the often poorer prediction compared to CNN approaches is a major point of criticism [5]. In contrast, feature extraction by means of CNN often delivers better results [5], but these are less comprehensible and cannot be compared in studies to the same extent; simultaneously, the often manual or semi-automatic segmentation is a major point of criticism here as well. Direct image analysis using deep learning does not require manual segmentation and is therefore probably the easiest to use in day-to-day business. Still in this case there is only limited understanding of how the prediction is made. In addition to these major differences between methods of image analysis, there are common limitations that hinder the clinical use of these systems.

Most studies put the focus of the analysis on a limited number of mutations instead of providing a comprehensive analysis of the genetic profile [9,10,14]. Furthermore, whilst some recent studies show excellent accuracies for the prediction of mutations [4,14,15], the study protocols entail high-profile, dedicated multiparametric MR imaging as imaging platforms. These protocols can be challenging to perform and, moreover, difficult to generalize for common clinical practice. Lastly, there has been a shift from manual segmentation [16] to semi-automatic [14] to fully automated algorithms [17]. Nevertheless, there is still a great number of recent publications that apply manual/semi-automatic segmentation, again impairing the generalization and clinical applicability of radiomics.

Hence, to address the above-mentioned limitations, the aim of this study was to establish a radiomics analysis based on fully automated segmentations and a simplified and robust MR imaging protocol to provide a comprehensive analysis of the genetic profile and grading of cerebral gliomas for everyday clinical use.

## 2. Methods

### 2.1. Ethics Statement

This study was performed in adherence to all of the guidelines defined by the approving institutional review board of the investigating hospital. Written informed consent was waived by the Institutional Review Board due to the retrospective nature of the study. Complete anonymization of all of the data was performed prior to their inclusion in the study.

### 2.2. Study Design and Cohort

**Figure 1:**
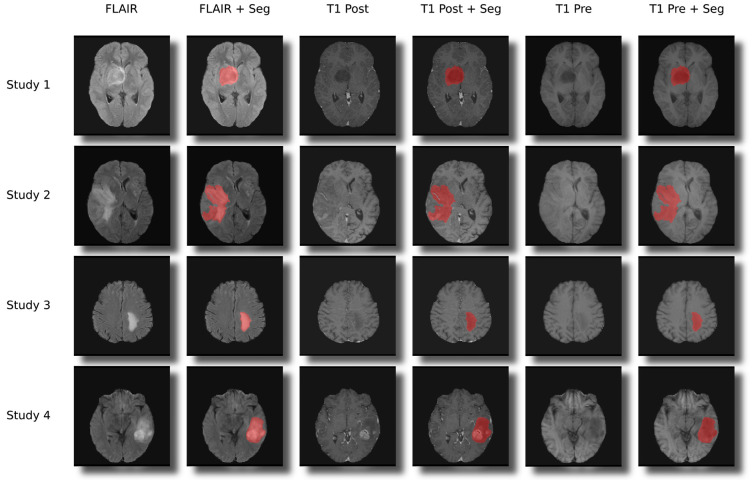
Examples for the segmentations and co-registration with other sequences.

**Figure 2:**
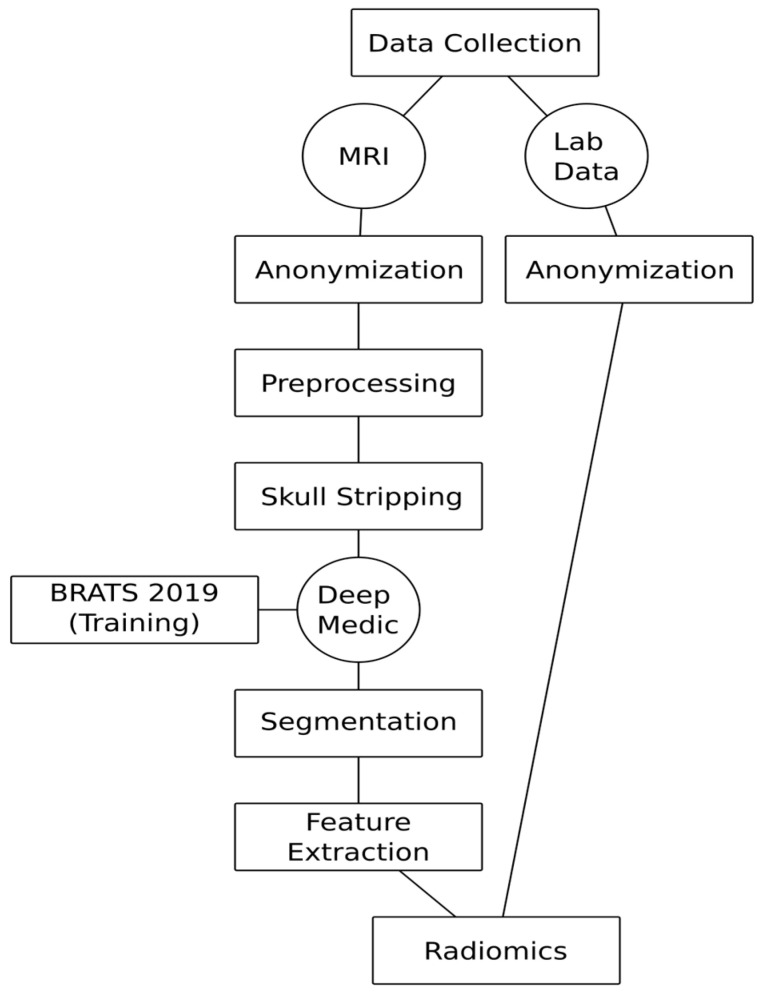
Automated virtual biopsy workflow.

217 MRI studies of 217 patients with histopathologically confirmed therapy naïve cerebral gliomas were included in the study. The collective comprised 28 low-grade gliomas (WHO 2) and 187 high-grade gliomas (WHO III + WHO IV). Two cerebral gliomas could not be conclusively classified and were excluded from the analysis. The distribution of genetic parameters on the training and test collective, as well as the age and sex distribution, are shown in Table 1. Exclusion criteria were previous brain surgery, pretreatment of the brain tumor or lack of histopathologic confirmation. The following sequences were included in the radiomics analysis: (1) FLAIR, (2) non-contrast T1-weighted sequence and (3) a contrast-enhanced T1-weighted sequence. All patients with incomplete MRI examinations were excluded from the study. The distribution of genetic parameters in the collective is shown in Table 1. In addition to the MRI data, clinical and laboratory parameters were included in the analysis (please refer to the clinical feature section). Prior to the inclusion of MRI data and clinical features, all data were anonymized. Subsequent to this, the preprocessing of the MRIs was performed, including co-registration and skull stripping. Thereafter, the data were used to segment the gliomas in an automated process using DeepMedic [18]. Example results of the automated segmentation are shown in Figure 1. Radiomics features were subsequently extracted from the segmented data and used in conjunction with the clinical features to predict the genetic profile and grading of cerebral gliomas using machine learning. The workflow is shown in Figure 2.

### 2.3. Magnetic Resonance Imaging

The MRI examinations were performed at a single center on assorted 1.5 T and 3 T MR-machines (MAGNETOM Symphony, MAGNETOM Sonata, MAGNETOM Avanto, MAGNETOM Aera, MAGNETOM Skyra) from a single vendor (Siemens Healthineers, Munich, Germany). The distribution among the different MR scanners is shown in Table 2, and the distribution of the train and test set among the different field strengths is shown in Table 3.

### 2.4. Preprocessing

Prior to executing the preprocessing, a brain extraction was performed to ensure an anonymized data set and to clean the image from unneeded structures. Due to its state-of-the-art performance, the publicly available HD-BET artificial neural network-based algorithm was used [19]. To spatially align the different series of a study, the SimpleITK extension SimpleElastix was used to co-register the series of a study as part of the preprocessing pipeline. A rigid registration, which is a form of a linear transformation, was carried out for all images using the post-contrast series as a fixed reference. The fully automated segmentation of the tumor regions was carried out using DeepMedic trained on the BraTS 2019 dataset, a Convolutional Neural Network (CNN) based algorithm [18].

### 2.5. Train Test Split

The full dataset was split into a training set and a test set with a ratio of 80:20, resulting in the number of studies in each set as stated in Table 1.

### 2.6. Clinical Features

The clinical features included gender, hemoglobin, c-reactive protein (CRP), sodium, calcium, total protein, platelets, aspartate aminotransferase (ASAT), lactate dehydrogenase (LDH) and bilirubin (total). The laboratory values were obtained within a time frame of 4 weeks prior to the MR examination. Listed laboratory data were available for all patients.

### 2.7. Radiomics Feature Extraction

To extract radiomics related features from the brain tumor images, the PyRadiomics package was used [20]. The extracted features comprise first-order statistics features, shape-based features, Gray Level Cooccurence Matrix (GLCM) features, Gray Level Run Length Matrix (GLRLM) features, Gray Level Size Zone Matrix (GLSZM) features, Neighbouring Gray Tone Difference Matrix (NGTDM) features and Gray Level Dependence Matrix (GLDM) features. Relevant features were additionally extracted from filter transformed images using Wavelet transformation, Laplacian of Gaussian (LoG) transformation, Local Binary Pattern 3D (LBP3D) transformation and Gradient transformation. In total, 1562 features were extracted for each series, adding up to a total of 4686 features per study.

### 2.8. Feature Selection

The number of extensive features leads to the necessity of selecting a subset of features with the highest predictive power to reduce the noise added by redundant features. For that purpose, the Boruta algorithm was utilized—a wrapper method that was combined with the Gradient Boosting algorithm XGBoost [21,22]. Subsets of features were selected with Boruta tuned to different thresholds, resulting in a relaxed selection process. Two different approaches of the Boruta Algorithm were used, one utilizing the permutation importance and the other based on the SHAP importance of the features in the underlying tree method. Table 4 shows the distribution of the three important features—volume, flatness and surface area—among the respective genetic profiles.

### 2.9. Parameter Optimization

The XGBoost parameters were tuned using the Tree-structured Parzen Estimator (TPE) sampler of the Optuna framework [23]. Each optimization run comprised 200 initial iterations with parameters picked randomly from a given parameter space before the final 2000 TPE steps were conducted. Each iteration of the optimization contained a bootstrapping-based cross-validation maximizing the logloss score of the validation data. The XGBoost model used with the Python API was initialized using the gbtree booster and the binary logistic objective within the parameter space given in Table 5.

### 2.10. Model Evaluation

For each label, the best result after the parameter optimization in terms of the AUC score of the validation set was picked. All model performance relevant metrics presented in this paper were calculated in a 1000fold bootstrapping cross-validation to obtain uncertainty intervals of the model performance. As the XGBoost algorithm returns an uncalibrated probability of a data point being of a given class, the sensitivity and specificity—depending on discrete outcomes—were calibrated using the geometric mean.

### 2.11. Validation of Segmentations

The performance of the DeepMedic segmentation Model was evaluated on 200 manually segmented patients. The cerebral gliomas were segmented manually by two radiology residents and inspected—and corrected—by the consensus of two radiology consultants. This resulted in a mean DICE score of 0.8082 ± 0.1321.

## 3. Results

**Figure 3:**
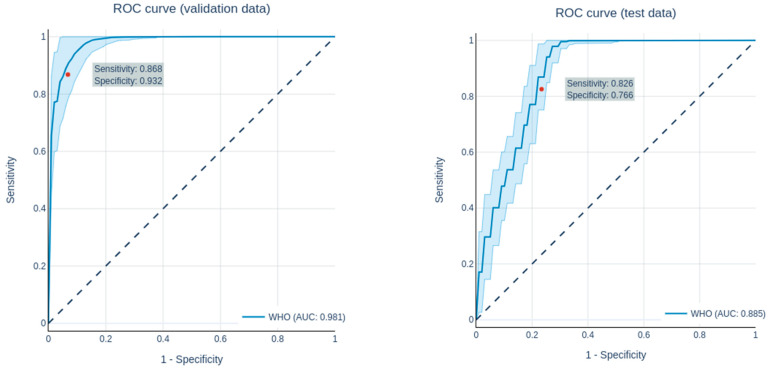
ROC curves of the prediction of the grading (LGG vs. HGG) in the validation data set (**left**) and the test data set **(right**).

**Figure 4:**
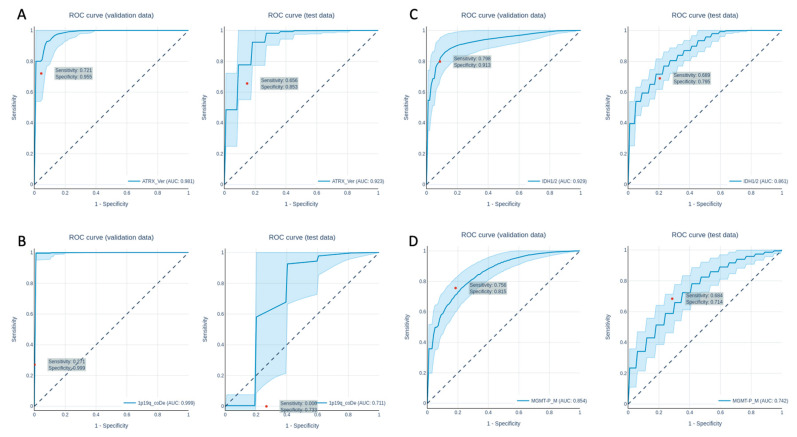
ROC curves of the prediction of the ATRX expression loss (**A**), the 1p19q co-deletion (**B**), the IDH1/IDH2 mutation (**C**) and the MGMT-status (**D**) in the validation data set (left) and the test data set (right).

Overall, all of the genetic parameters could be predicted with a good performance. The ROC curves of the predictions are shown in Figure 3 and Figure 4, while the AUC values, accuracy, precision, sensitivity and specificity of the train, validation and test set for each genetic parameter and grading (LGG vs. HGG) are shown in Table 6 and Table 7. All predictions were compared with the prediction of a dummy classifier in order to avoid possible errors due to an unbalanced collective.

The results of the dummy classifier are interpreted as the best results that can be achieved without training a model by guessing the prediction based on the class imbalance. A classifier was chosen that predicts the data by randomly classifying each data point as positive or negative while taking a possible class imbalance into account (stratified). Overall, all predictions of the dummy classifier were very close to an AUC of 0.5 (0.497, 0.498, 0.513, 0.500, 0.503), and, therefore, no capability of separating the classes from each other was observed.

### 3.1. Radiomics Analysis to Predict Grading

Grading in this study was limited to the clinically highly relevant differentiation between LGG (WHO 2) and HGG (WHO 3 + 4). Following the feature selection, 56 features were selected for the predictions. All in all, with an AUC of 1.0 ± 0.000/0.981 ± 0.015/0.885 ± 0.02(training/validation/test), the network showed a good performance in differentiating between LGG and HGG. The performance in predicting grading in the validation and test datasets are shown in Table 6, while the ROC curves for the predictions of the validation and test datasets are shown in Figure 3.

### 3.2. Radiomics Analysis to Predict the Genetic Profile

The analysis of the genetic parameters showed overall good to very good results. In detail, the network performed well in predicting the ATRX expression with an AUC of 1.0 ± 0.000/0.979 ± 0.028/0.923 ± 0.045(training/validation/test). A total of 18 features were used for prediction, which were selected by feature selection. The performance to predict the genetic parameters of the validation and test datasets are shown in Table 7. The ROC curves for the predictions of the validation and test datasets are shown in Figure 4.

For the prediction of the 1p19q co-deletion, the network showed a heterogeneous performance, with an AUC of 1.0 ± 0.000/0.999 ± 0.005/0.711 ± 0.128 (training/validation/test). The ROC curves for validation and test dataset predictions are plotted in Figure 4B. The predictions were based on 11 features, which were selected by feature selection.

The networks performance in predicting IDH1/2 mutation was quite good, with an AUC of 1.0 ± 0.000/0.929 ± 0.042/0.861 ± 0.023 (training/validation/test). The ROC curves for the validation and test dataset predictions are shown in Figure 4C. For predictions, a number of 36 features were used. For the prediction of the MGMT methylation status, the network performed well, with an AUC of 1.0 ± 0.000,0.854 ± 0.046, 0.742 ± 0.050 (training/validation/test) using 25 features, which were previously selected using feature selection. The ROC curves for the validation and test dataset predictions are shown in Figure 4D.

## 4. Discussion

In recent years, radiomics-based tissue characterization has demonstrated its potential for non-invasive prediction of the genetic profile and grading in cerebral gliomas using multiparametric MRI [9,17,24]. The benefits of this innovative tissue characterization form include its non-invasive nature and whole-tumor assessment when compared to focal stereotactic biopsy sampling, with an associated mortality rate of 2.8% [3]. Nevertheless, a number of challenges have remained: the majority of published studies are limited to the prediction of a genetic parameter and/or grading, lacking the convenience and comparability to an extensive invasive workup [9,25,26,27]. Additionally, previous studies are predominantly based on manual or semi-automatic segmentation methods, which significantly limits reproducibility and inter-study comparability [28]. Furthermore, these studies generally require a complex MRI protocol and standardized sequences and are mainly based on specific field strengths or specific scanners, impeding the transition from local unicenter radiomics-based tumor assessment to a more universal approach.

Hence, the main aim of our study was to address the majority of these limitations to prospectively ease the transition of radiomics-based non-invasive tissue sampling into clinical practice. Thus, our study comprised a collective of 217 patients acquired on seven different MRI scanners, entailing both 1.5 as well as 3 Tesla scanner systems. The radiomics analysis was based on a basic imaging protocol consisting of three universally applied sequences: a FLAIR, a non-contrast and a contrast-enhanced T1-weighted sequence with variable imaging parameters. Previous publications indicate a direct correlation of the performance power of radiomics to the homogeneity and complexity of MR protocols [4]. Nevertheless, neither homogeneous imaging parameters nor complex protocols reflect the global clinical approach to MR imaging. Thus, to understand whether radiomics-based tumor assessment can be applied in a broader scope and more universal approach, we took a potential loss in predictive power into account.

To account for reproducibility and to remove potential human bias or influence of any kind, we applied DeepMedic for the automated segmentation of cerebral gliomas, which had been pretrained with the BraTS 2019 dataset [18,29,30,31]. On this basis, feature selections of radiomics parameters were performed and used for training machine learning algorithms. During the feature selection, gender and laboratory parameters dropped out because of their poor predictive power, so only imaging parameters were used to train the final model. While gender is certainly a risk factor for cerebral gliomas [32], this feature does not appear to be relevant for differentiating genetic parameters and grading in our study collective. No data on association with grading and genetic parameters are available for the laboratory parameters used. Nevertheless, there are various studies on the improvement of predictive power in a variety of other tumor entities by laboratory parameters [33], so we wanted to test the predictive power for our study setting.

One of the most compelling challenges for radiomics-based tumor assessment is the comparability to the extensive invasive work up, as the majority of previously published studies focused on single parameter analyses [9,14]. Therefore, we included an extensive work up including the differentiation of high-grade versus low-grade gliomas and ATRX, IDH1/2, 1p19q, MGMT in our analysis. The clinical management of a patient is crucially dependent on the genetic profile and the differentiation of LGG vs. HGG [34,35]. For this purpose, in this study, the network was able to differentiate LGG from HGG with an AUC of 1.0 ± 0.000/0.981 ± 0.015/0.885 ± 0.02 (train/validation/test), yielding very good results, which are on a similar level or even better compared to other studies with more complex protocols and more homogeneous collectives [8,36]. Overall, good results were obtained in predicting the genetic parameters, although these varied depending on the type of genetic parameter, as described previously in the literature [4,9,27,37].

For the predictions of ATRX, IDH1/2, 1p19q and MGMT, this resulted in an AUC (train/validation/test) of 1.0 ± 0.000/0.979 ± 0.028/0.923 ± 0.045 (ATRX), 1.0 ± 0.000/0.929 ± 0.042/0.861 ± 0.023 (IDH1/2), 1.0 ± 0.000/0.999 ± 0.005/0.711 ± 0.128 (1p19q) and 1.0 ± 0.000, 0.854 ± 0.046, 0.742 ± 0.050 (MGMT). Our results for the grading and prediction of ATRX and IDH1/2 are comparable and equally good in comparison to a previously published study on a highly-complex 18F-FET-PET/MRI study involving MR Fingerprinting as a new relaxometry technique [4]. This could be due to the fact that, in our current study, the collective was significantly larger. Thus, the predictive power of the ATRX expression loss and IDH1/2 mutation is similar to or higher than other recent publications, although fewer sequences were used in our study [9,27,38].

However, following the excellent accuracies for prediction of 1p19q co-deletion and IDH1/2 mutation, our results reveal a substantial drop as well as noticeably strong variation in the prediction of the 1p19q co-deletion in our test and validation collective. This is probably due to the fact that the 1p19q co-deletion is the least present mutation in the collective. Hence, this issue should be further investigated in a larger collective, entailing more patients with the 1p19q co-deletion.

Overall, the small number of patients with a 1p19q co-deletion in our collective reduces the predictive power. To address this, we performed a 1000fold bootstrapping cross-validation inside the hyperparameter optimization on all predictions. Overall, however, this remains a limitation, particularly regarding the 1p19q co-deletion, which can only be fully addressed in larger follow-up studies.

Furthermore, a substantial drop from the test to the validation collective is often observed in radiomics studies [27]. In our study, this might have been caused by the very heterogeneous collective consisting of scans from seven different MRI scanners. While some might see this as a limitation, it is also an advantage, as it improves transferability. Nevertheless, this can also be a sign of overfitting. To avoid overfitting, we used early stopping of the training process based on the validation score (early_stopping = 10), restricted the tree depth to a max of 6 and used a cross-validation inside the hyperparameter optimization.

Alternatively, according to the literature, additional T2 mapping may significantly increase the predictive power for the 1p19q co-deletion [4]. Lastly, in line with current literature, our network only enabled a moderate prediction of the MGMT mutation with AUCs of 0.742, hence, deeming the prediction of this parameter as non-clinical [39].

Overall, despite our promising results, our study is not free of limitations. One limitation is that, while our study was performed on different MRI scanners with 1.5 and 3 T, there is a lack of diversity from different manufacturers, which may alter the results. In addition, recent studies have shown that different methods of data normalization can improve the robustness of radiomics predictions in a heterogeneous collective, a technique that may further increase the efficiency of our workflow in the future [40]. Further limitations are the retrospective setting and the partly small sample size, e.g., for the 1p19q co-deletion, which was addressed by our extensive validation using the train/validation/test collective. These issues could be easily addressed in a follow-up multi-center setup using the proposed fully automated workflow.

Finally, a biopsy cannot be waived if a requirement to use the network is that a glioma is present. Although this is the largest group of primary cerebral neoplasms, other rarer entities must be differentiated from this. Therefore, further networks should be used to differentiate important differential diagnoses such as cerebral lymphoma. Although a biopsy in the setting of initial diagnosis of cerebral neoplasm cannot be avoided without the help of additional networks, the prediction of the genetic profile could help to early detect mutations from low-grade gliomas to higher-grade gliomas in follow-up examinations.

## 5. Conclusions

In summary, the present study demonstrated that a fully automated prediction of grading and the genetic profile of cerebral gliomas could be performed with our proposed method using a simplified MRI protocol that is robust to variations in scanner systems, imaging parameters and field strength.

## Figures and Tables

**Table 1:** Initial patient collective: Distribution of the training, testing and total cohort of the respective genetic parameters, age and gender. A positive label is defined as having an IDH1/2 mutation, an ATRX expression loss, a 1p19q co-deletion and a positive MGMT methylation status. For the WHO label, LGG is defined as positive and HGG as negative. The gender distribution is expressed as the percentage of females in the collective.

Label	Total (pos/neg)	Train (pos/neg), %Female, Mean Age + SD	Test (pos/neg) %Female, Mean Age + SD
MGMT	164 (81/83)	131 (65/66), 45.0%, 57.0 ± 14.3 years	33 (16/17), 30.3%, 60.6 ± 13.1 years
IDH1/2	145 (34/111)	116 (27/89), 37.9%, 55.5 ± 16.9 years	29 (7/22), 41.4%, 50.2 ± 17.8 years
1p19q	30 (5/25)	24 (4/20), 45.8%, 45.3 ± 14 years	6 (1/5), 33.3%, 56.7 ± 6.9 years
WHO	215 (28/187)	172 (22/150), 41.9%, 56.0 ± 16.2 years	43 (6/37), 41.9%, 52.6 ± 15.4 years
ATRX	67 (13/54)	53 (10/43), 32.1%, 56.8 ± 13.3 years	14 (3/11), 64.3%, 56.9 ± 9.5 years

**Table 2:** Distribution of examinations among MR scanners (all scanners are from Siemens Healthineers).

Scanner	1.5 T Magnetom Aera	3 T Magnetom Skyra	1.5 T Magnetom Espree	3 T Biograph_mMR	1.5 T Magnetom Avanto	1.5 T Magnetom Sonata	1.5 T Magnetom Symphony
*n*	68	57	35	19	19	12	10

**Table 3:** Distribution of the train and test among magnetic field strength.

Labels	Magnetic Field Strength	Genetic Profile				
		MGMT	IDH1/2	ATRX	1p19Q	WHO_HIGH
0 (train)	1.5	45	53	21	11	15
	3.0	21	36	22	9	7
1 (train)	1.5	38	14	6	2	76
	3.0	27	13	4	2	74
0 (test)	1.5	14	15	7	3	3
	3.0	3	7	4	2	3
1 (test)	1.5	10	4	2	0	26
	3.0	6	3	1	1	11

**Table 4:** Lesion-specific features for positive and negative samples and the Bonferroni corrected Mann-Whitney U statistics grouped by label.

	MGMT	WHO	IDH 1/2	1p19q	ATRX
Volume	neg: 9.9 × 10^4^ ± 5.5 × 10^4^	neg: 1 × 10^5^ ± 5.8 × 10^4^	neg: 9.9 × 10^4^ ± 5.8 × 10^4^	neg: 9.1 × 10^4^ ± 7 × 10^4^	neg: 1.1 × 10^5^ ± 6.5 × 10^4^
	pos: 1.1 × 10^5^ ± 6.3 × 10^4^	pos: 5.8 × 10^4^ ± 6.8 × 10^4^	pos: 9.1 × 10^4^ ± 8.2 × 10^4^	pos: 1 × 10^5^ ± 1.2 × 10^5^	pos: 9.2 ×10^4^ ± 6.4 × 10^4^
	*p* = 0.340	*p* < 0.0001	*p* = 0.070	*p* = 0.434	*p* = 0.194
Flatness	neg: 0.59 ± 0.12	neg: 0.58 ± 0.12	neg: 0.59 ± 0.13	neg: 0.57 ± 0.13	neg: 0.59 ± 0.13
	pos: 0.58 ± 0.12	pos: 0.65 ± 0.1	pos: 0.63 ± 0.11	pos: 0.65 ± 0.091	pos: 0.62 ± 0.1
	*p* = 0.276	*p* = 0.002	*p* = 0.013	*p* = 0.110	*p* = 0.177
Surface Area	neg: 2.1 × 10^4^ ± 9.9 × 10^3^	neg: 2.2 × 10^4^ ± 1.1 × 10^4^	neg: 2.1 × 10^4^ ± 1.1 × 10^4^	neg: 2.1 × 10^4^ ± 1.5 × 10^4^	neg = 2.2 × 10^4^ ± 1.2 × 10^4^
	pos: 2.2 × 10^4^ ± 1.2 × 10^4^	pos: 1.3 × 10^4^ ± 1.4 × 10^4^	pos: 1.8 × 10^4^ ± 1.6 × 10^4^	pos: 2.2 × 10^4^ ± 2.4 × 10^4^	pos: 1.9 × 10^4^ ± 1.2 × 10^4^
	*p* = 0.358	*p* < 0.0001	*p* = 0.012	*p* = 0.348	*p* = 0.177

**Table 5:** Parameter space used for hyperparameter optimization with the Tree-structured Parzen Estimator (TPE).

Hyperparameter	
n_estimators	[100, 1500] stepsize: 100
max_depth	[1, 6] stepsize: 1
learning_rate	[0.05, 0.03] stepsize: loguniform
gamma	[0, 20] stepsize: uniform
min_child_weight	[1, 20] stepsize: 1
subsample	[0.5, 1.0] stepsize: 0.05
colsample_bytree	[0.1, 1.0] stepsize: 0.05
reg_landa	[1 × 10^−8^, 1.0] stepsize: loguniform
reg_landa	[1 × 10^−8^, 1.0] stepsize: loguniform

**Table 6:** Performance in predicting the grading.

Dataset	AUC	Accuracy	Precision	Sensitivity	Specificity
Grading-Validation	0.981 ± 0.015	0.925 ± 0.042	0.685 ± 0.168	0.868 ± 0.035	0.932 ± 0.048
Grading-Test	0.885 ± 0.021	0.774 ± 0.032	0.369 ± 0.042	0.826 ± 0.035	0.766 ± 0.040

**Table 7:** Performance in predicting the genetic parameters.

Dataset	AUC	Accuracy	Precision	Sensitivity	Specificity
ATRX-Validation	0.981 ± 0.028	0.914 ± 0.048	0.844 ± 0.193	0.721 ± 0.089	0.955 ± 0.060
ATRX-Test	0.923 ± 0.045	0.810 ± 0.066	0.593 ± 0.174	0.656 ± 0.059	0.853 ± 0.086
1p19q-Validation	0.999 ± 0.005	0.889 ± 0.032	0.521 ± 0.497	0.271 ± 0.260	0.999 ± 0.012
1p19q-Test	0.711 ± 0.128	0.611 ± 0.094	0.000 ± 0.000	0.000 ± 0.000	0.733 ± 0.113
IDH1/2-Validation	0.929 ± 0.042	0.888 ± 0.045	0.751 ± 0.131	0.798 ± 0.087	0.913 ± 0.059
IDH1/2-Test	0.861 ± 0.023	0.769 ± 0.053	0.544 ± 0.113	0.689 ± 0.095	0.795 ± 0.091
MGMT-Validation	0.854 ± 0.046	0.786 ± 0.044	0.807 ± 0.076	0.756 ± 0.094	0.815 ± 0.091
MGMT-Test	0.742 ± 0.050	0.699 ± 0.046	0.705 ± 0.077	0.684 ± 0.125	0.714 ± 0.117

## Data Availability

The data presented in this study are available on request from the corresponding author. The data are not publicly available for data protection reasons.
